# Mechanisms and Therapeutic Targets of Ischemia—Reperfusion Injury in Stroke: A Narrative Review Focusing on Blood—Brain Barrier Dysfunction

**DOI:** 10.3390/brainsci16050469

**Published:** 2026-04-27

**Authors:** Suqin Guo, Rui Liu, Si Cheng, Xia Liu, Jianping Wu

**Affiliations:** 1 School of Chemistry, Chemical Engineering and Life Sciences, Wuhan University of Technology, Wuhan 430070, China; guosq678@whut.edu.cn; 2 School of Basic Medical Sciences, Hubei University of Medicine, Shiyan 440070, China; 20110511@hbmu.edu.cn; 3 Beijing Tiantan Hospital, Capital Medical University, Beijing 100070, China; sicheng@ncrcnd.org.cn; 4 China National Clinical Research Center for Neurological Diseases, Beijing 100070, China; 5 Beijing Key Laboratory of Drug and Device Research and Development for Cerebrovascular Diseases, Beijing 100070, China

**Keywords:** ischemic stroke, blood–brain barrier, ischemia–reperfusion injury, neuroprotection, therapeutic strategies

## Abstract

**Highlights:**

**What are the main findings?**

- BBB disruption in stroke I/R injury follows a distinct spatiotemporal hierarchy.
- A comprehensive treatment framework covering three major strategies has been proposed.

**What are the implications of the main findings?**

- Therapeutic strategies must be phase-specific and time-limited rather than continuously blocking a single pathway.
- This explains why many intervention measures that have shown efficacy in animal models have failed in clinical trials.

**Abstract:**

Ischemic stroke remains a leading cause of death and disability worldwide. While thrombolysis and endovascular thrombectomy are current mainstays of treatment, their clinical efficacy is often undermined by ischemia–reperfusion injury (I/R). This injury induces secondary brain damage, primarily via disruption of the blood–brain barrier (BBB). No approved therapies directly target BBB protection. This review reinterprets the pathophysiological mechanism of BBB disruption after stroke through a dynamic spatiotemporal framework. The pathological cascade reaction is clearly divided into two core stages: the ischemic phase is mainly driven by energy failure and calcium overload; the reperfusion phase is further divided into four consecutive and progressive sub-stages, namely, oxidative stress burst, amplification of inflammatory response, matrix metalloproteinase 9 (MMP-9)-mediated barrier degradation and programmed cell death. This review critically assesses current therapies and identifies major clinical translation gaps, including a temporal mismatch between preclinical and clinical windows, unacceptable toxicity, lack of durable efficacy and biphasic effects. Matching specific interventions to the different pathophysiological stages of blood–brain barrier disruption is essential for optimizing clinical outcomes.

## 1. Introduction

### 1.1. Background

Stroke, also termed acute cerebrovascular accident, represents a major global public health burden, characterized by high incidence, mortality, disability and recurrence rates. Epidemiological evidence indicates that stroke is the leading cause of death in China [1]. The rising incidence in younger adults further increases the societal and economic burden.

**Figure 1:**
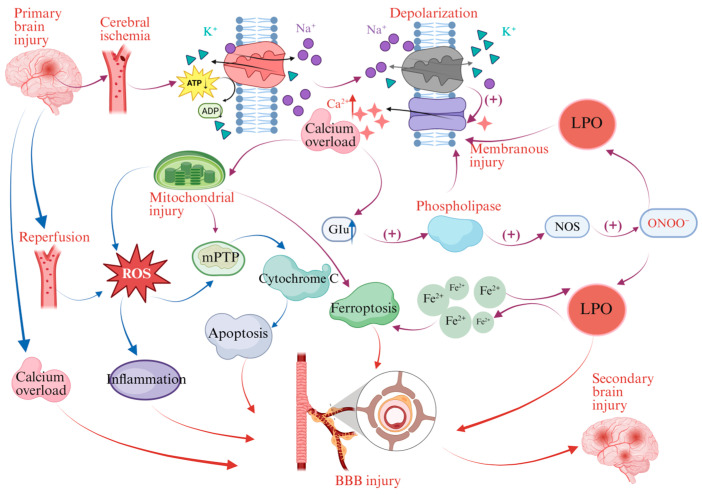
Complex molecular mechanisms of BBB disruption during CIRI. This schematic diagram systematically presents multiple pathophysiological processes after cerebral ischemia, such as oxidative stress response, ferroptosis response, inflammatory response, membrane damage and apoptosis. These processes act in concert to compromise the integrity and function of the BBB. (Abbreviations: ROS: reactive oxygen species; NOS: nitric oxide synthase; ONNO^−^: peroxynitrite anion; mPTP: mitochondrial permeability transition pore; Glu: glutamic acid; LPO: lipid peroxidation; (+): represents promotion. Regulatory and symbolic indicators: → indicates pathological cascade and sequential progression).

Ischemic stroke typically results from vascular occlusion, which triggers energy failure, calcium overload, oxidative stress and mitochondrial dysfunction, leading to neuronal injury [2]. Although revascularization therapies, such as thrombolysis and thrombectomy, can restore blood flow, some patients experience CIRI. This condition is characterized by BBB disruption, oxidative stress and neuroinflammation [3,4]. CIRI involves multiple mechanisms, including reactive oxygen species (ROS), overproduction, acidosis, calcium dysregulation and activation of inflammatory and regulated cell death pathways, which collectively worsen outcomes [5]. These processes collectively exacerbate BBB disruption, neuroinflammation and apoptosis (Figure 1). The lack of effective targeted interventions makes CIRI prevention and treatment a critical unmet need in stroke care.

During ischemic stroke, abrupt vascular occlusion and loss of cerebral blood flow trigger rapid endothelial and BBB alterations. Contraction of brain microvascular endothelial cells (BMECs) causes tight junction (TJ) proteins to translocate from the membrane to the cytosol, disrupting barrier integrity and increasing BBB permeability. This allows solutes, cytokines and immune cells to infiltrate the brain, initiating further inflammation and tissue injury [6].

### 1.2. Rationale for BBB-Focused Intervention

Damage to the BBB following reperfusion therapy often leads to inevitable secondary injury [3]. Despite the diverse mechanisms, most neuroprotective concepts focus on activating endogenous protective pathways and limiting secondary injury [7]. Early diagnosis and timely intervention are critical determinants of functional outcome following ischemic stroke. Although recanalization therapy is feasible in clinical practice, its ability to alleviate BBB disruption remains limited [8]. Therapeutic strategies specifically aimed at preserving BBB integrity and promoting its repair hold significant promise for improving neurological recovery.

### 1.3. Literature Search Strategy

For this narrative review, we searched PubMed and Web of Science from inception to April 2026 using combinations of keywords including “cerebral ischemia–reperfusion injury,” “blood–brain barrier,” “oxidative stress,” “calcium overload,” “inflammation,” “MMPs,” “programmed cell death,” “apoptosis,” “pyroptosis,” “ferroptosis,” “clinical trial,” and “stroke.” We prioritized peer-reviewed original articles, systematic reviews, and phase II–IV clinical trials. Reference lists of retrieved articles were manually screened for additional relevant studies. Given the narrative nature of this review, we did not perform a formal quality assessment or meta-analysis but instead focused on synthesizing mechanistic insights and translational lessons across the literature.

### 1.4. What Is New in This Review?

Unlike previous reviews that describe BBB injury mechanisms as parallel, static events, this review introduces a Temporal-Stage Therapeutic Matching Framework. We propose that the pathological cascade follows an ordered, time-dependent sequence, and that clinical failures often result from a mismatch between a drug’s mechanism of action and the spatiotemporal window of BBB pathology. This framework is elaborated in the Discussion, where we derive specific translational lessons and future directions.

## 2. Structure and Function of the BBB

The BBB is a multicellular structure composed of brain microvascular endothelial cells (BMECs), pericytes, astrocytes and a basement membrane [9]. It maintains ion homeostasis and the immune microenvironment required for neuronal activity, while preventing entry of peripheral toxins and pathogens [10]. Disruption of the BBB is implicated in various CNS diseases and often serves as a trigger for secondary injuries such as hemorrhage and cerebral edema [11].

BMECs form the inner layer of microvessels, connected by tight and adherens junctions to establish selective permeability. Pericytes wrap BMECs to regulate vascular stability and permeability via signaling pathways, while the basement membrane provides structural support and filtration [12]. The endothelial basement membrane close to endothelial cells and the parenchymal basement membrane adjacent to astrocyte terminals are separated by pericytes [13].

Astrocytes maintain extracellular ion balance, regulate neurotransmitter turnover and release neurotrophic factors [14]. They maintain extracellular ion balance, regulate neurotransmitter turnover, modulate cerebral blood flow and release neurotrophic factors. Reactive astrocyte proliferation and glial scar formation are adaptive responses after ischemic stroke, supporting neuronal survival and promoting plasticity. Dysregulated astrocytes may exacerbate inflammation, form incomplete glial scars and contribute to toxic microenvironments that impair neuronal recovery [15].

Microglia are the resident immune cells of the CNS [16]. Following cerebral ischemia, microglia are rapidly activated and polarize into either pro-inflammatory (M1) or anti-inflammatory (M2) phenotypes [17]. M1-polarized microglia secrete pro-inflammatory mediators, including tumor necrosis factor-α (TNF-α), IL-1β, IL-6, IL-18, IL-23, inducible nitric oxide synthase (iNOS) and matrix metalloproteinases (MMPs), such as MMP-9 and MMP-3, collectively contributing to BBB disruption. M2-polarized microglia release anti-inflammatory cytokines, such as IL-10 and various growth factors, thereby supporting angiogenesis and tissue repair [18]. Their roles in oxidative stress, calcium overload and BBB dysfunction are discussed in subsequent sections.

## 3. The Mechanism of I/R on BBB

During the ischemic phase (minutes to hours), energy depletion, intracellular calcium overload and glutamate-mediated excitotoxicity constitute the principal initiating pathological mechanisms, triggering early disruption of tight junctions and cytotoxic swelling of endothelial cells. In the subsequent reperfusion phase, a burst of ROS production, inflammatory activation, MMP-mediated degradation and various forms of programmed cell death act synergistically to amplify and sustain BBB damage; these spatiotemporally dynamic interactions collectively result in increased BBB permeability and the development of vasogenic brain edema (Table 1).

### 3.1. Early Injury Stage

#### 3.1.1. Energy Depletion, Calcium Overload and Initial Injury

CIRI begins with an energy crisis. Disordered energy metabolism after brain injury is the primary driver of secondary injury. Interruption of blood flow causes brain cells to experience a serious energy crisis (ATP depletion). This forces them to shift from aerobic to inefficient anaerobic metabolism, resulting in lactic acid accumulation and acidosis. Mitochondrial dysfunction further worsens this condition, leading to Na^+^/K^+^-ATPase inactivation, cell swelling and calcium influx [29]. The astrocyte–neuron lactate shuttle (ANLS) pathway is disrupted, preventing neurons from obtaining sufficient lactate for energy and thereby exacerbating the energy shortage [30]. This imbalance magnifies the chain reaction of excitotoxicity and diffuse cortical depolarization, leading to more extensive cell death [31].

Ca^2+^ overload triggers toxic cascades [32,33,34]:

- Activation of Ca^2+^-dependent enzymes (phospholipases, proteases, nitric oxide synthase);
- Calpain-mediated degradation of cytoskeletal and pro-apoptotic proteins;
- Mitochondrial permeability transition pore (mPTP) opening;
- Cytochrome C release and ATP depletion;
- Excess ROS production and nuclear factor kappaB (NF-κB)-driven neuroinflammation.

Ischemia induces increased glutamate release from neurons and astrocytes, driving excitotoxicity. This process, together with oxidative stress, impairs mitochondrial dynamics, increasing the levels of fragmented mitochondria and generating a large number of dysfunctional mitochondria with reduced ATP production and increased ROS leakage [32].

#### 3.1.2. Oxidative Stress Burst and ROS-Mediated Injury

Upon reperfusion, the abrupt reintroduction of oxygen into compromised tissue precipitates a rapid and robust burst of ROS, representing a defining pathogenic event of the reperfusion phase and a central mechanistic contributor to BBB disruption [35]. ROS directly damage BMECs, inducing lipid peroxidation, protein oxidation and DNA damage, ultimately triggering endothelial cell death and BBB breakdown [36].

The kallikrein–kinin system is an additional source of ROS. Bradykinin levels increase during I/R and activate NADPH oxidase via B2 receptor-mediated PLC–PKC and Ca^2+^ pathways. This leads to phosphorylation and redistribution of TJ proteins [37].

ROS also induce matrix metalloproteinase expression and activation, degrading TJ proteins and basement membrane components. ROS stimulate the NF-κB pathway, promoting release of TNF-α, IL-1β and IL-6 and upregulating adhesion molecules on endothelial cells, facilitating leukocyte adhesion and transmigration [38].

Spatiotemporally, the ROS burst occurs within minutes of reperfusion onset and primarily targets endothelial mitochondria and membrane lipids. This phase sets the stage for subsequent inflammatory amplification [35].

#### 3.1.3. Inflammatory Response and Immune Amplification

Following the initial ROS burst, a secondary wave of injury ensues, driven by robust inflammatory activation [39]. After ischemia, activation of microglia and astrocytes increases the production of cytokines, chemokines, MMPs and vascular endothelial growth factor (VEGF) in ischemic brain tissue. These factors degrade TJ proteins, alter endothelial cytoskeletal structure, upregulate adhesion molecules, and promote neutrophil and monocyte infiltration across the BBB [40].

The principal inflammatory cytokines (TNF-α, IL-1β, IL-6, IL-17) induce elevated vascular permeability and maintain inflammation. TNF-α and IL-1β also induce the production of MMP-2 and MMP-9, which degrade intercellular adhesion molecules and extracellular matrix (ECM) components [41]. Inflammasome activation (e.g., NLRP3) and complement system (C3a, C5a) pathways further amplify inflammation, creating a self-perpetuating cycle of BBB injury [42,43].

Oxidative stress and inflammation are mutually reinforcing during this phase [44]. As noted in Section 3.1.2, ROS activate NF-κB and MAPK pathways, upregulating pro-inflammatory cytokines. Cytokines, such as TNF-α and IL-1β, stimulate ROS production via NADPH oxidase and mitochondrial pathways [45,46]. This positive feedback loop sustains BBB injury and supports the use of combined antioxidant and anti-inflammatory therapy as a rational strategy for CIRI.

### 3.2. Matrix Degradation Stage

MMPs are zinc-dependent endopeptidases that degrade ECM components, modulating tissue remodeling and the inflammatory response [47]. MMP-9 is particularly important in acute ischemic stroke, as its early upregulation correlates with hemorrhagic transformation risk and poor neurological outcomes [48]. Sources of MMP-9 include infiltrating neutrophils, activated microglia, astrocytes and BMECs [49].

MMP-9 rapidly increases in the acute phase and is the main substance that destroys the BBB. MMP-2 and MMP-9 are rapidly induced and activated, causing degradation of the BBB basement membrane components (type IV collagen, laminin, fibronectin) and cleavage of TJ proteins, such as occludin, claudin-5 and ZO-1 [50,51]. This also raises the risk of hemorrhagic transformation. During the reperfusion recovery period, MMP2 and MMP9 levels rise again. These MMPs degrade the ECM, promoting the migration of vascular endothelial cells and the formation of new blood vessels, which can help repair damaged blood vessels [51]. This suggests that MMPs may exert dual biological effects in the pathogenesis and progression of stroke.

MMP activity not only compromises BBB structural integrity but also facilitates leukocyte infiltration, amplifies inflammation and generates bioactive ECM fragments that further stimulate immune responses [52].

### 3.3. Programmed Cell Death Stage

#### 3.3.1. Apoptosis

Apoptosis is a tightly regulated, non-inflammatory form of programmed cell death that eliminates damaged or unnecessary cells without inducing inflammation [53]. During CIRI, apoptosis affects neurons, astrocytes, endothelial cells and oligodendrocytes, driven by intrinsic (mitochondrial), extrinsic (death receptor) and endoplasmic reticulum stress pathways [54].

The intrinsic pathway is triggered by mitochondrial outer membrane permeabilization (MOMP) in response to Ca^2+^ overload, oxidative stress or DNA damage [55,56]. The extrinsic pathway involves death receptor activation (Fas, TNFR1) by their ligands (FasL, TNF-α). This leads to the death-inducing signaling complex (DISC) and caspase-8 activation [57,58]. Crosstalk between extrinsic and intrinsic pathways occurs via caspase-8-mediated cleavage of Bid to tBid, which enhances mitochondrial permeabilization [59]. Endoplasmic reticulum (ER) stress in CIRI is triggered by ischemia-induced disruption of Ca^2+^ homeostasis and protein misfolding, activating the CHOP/PERK/ATF6/IRE1 pathway, which can induce apoptosis via caspase-12 activation [60].

#### 3.3.2. Pyroptosis

Pyroptosis is a pro-inflammatory form of programmed cell death characterized by rapid plasma membrane rupture, release of pro-inflammatory intracellular contents and strong immune activation. It is driven by inflammasome activation and caspase-mediated cleavage of gasdermin proteins, especially gasdermin D (GSDMD) [61].

In CIRI, damage-associated molecular patterns (DAMPs), such as ATP, HMGB1 and mitochondrial DNA, activate pattern recognition receptors (PRRs), including NLRP3, AIM2 and NLRC4 [62]. This triggers the assembly of inflammasome complexes, the recruitment of adaptor protein ASC and the subsequent activation of caspase-1. Activated caspase-1 cleaves GSDMD, releasing its N-terminal pore-forming fragment, which oligomerizes and inserts into the plasma membrane. This pore formation induces an osmotic imbalance, resulting in cell swelling, lytic cell death (pyroptosis) and the concomitant release of the pro-inflammatory cytokines IL-1β and IL-18 [63,64].

#### 3.3.3. Ferroptosis

Ferroptosis is an iron-dependent form of programmed cell death distinguished by excessive lipid peroxidation and plasma membrane damage. It is initiated by intracellular iron accumulation, lipid ROS generation and impaired antioxidant defenses, particularly the loss of GPX4 activity [65,66].

During CIRI, iron metabolism is disrupted through multiple mechanisms, including upregulation of transferrin receptor 1 (TfR1), downregulation of ferroportin and ferritin degradation. These alterations drive a pathological expansion of the intracellular labile iron pool [67]. Excess of Fe^2+^ participates in the Fenton reaction to generate highly reactive free radicals, which attack polyunsaturated fatty acids (PUFAs) located within the phospholipid bilayer of cellular membranes, thereby initiating the lipid peroxidation chain reaction [62].

The ACSL4/LPCAT3/ALOX15 pathway critically regulates ferroptosis signaling, catalyzing PUFA acylation into phosphatidylethanolamine and driving their peroxidation, thereby amplifying lipid peroxidation [68]. This cascade not only triggers neuronal ferroptosis but also compromises vascular endothelial integrity, increasing BBB permeability and facilitating inflammatory cell infiltration.

## 4. Therapeutic Strategies for Protecting the BBB in CIRI

**Figure 2:**
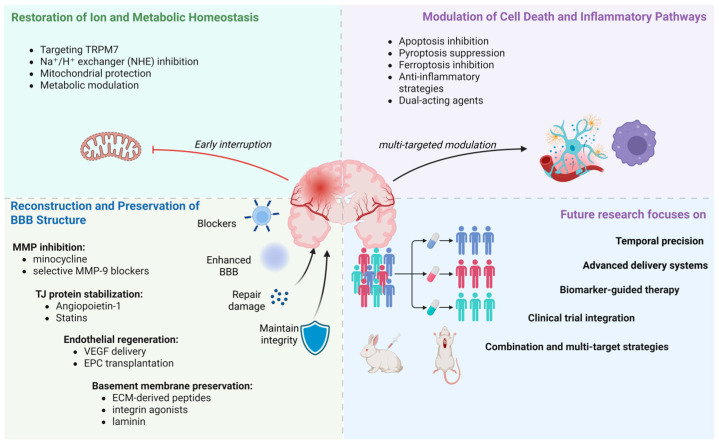
Multifaceted therapeutic approaches targeting BBB injury in cerebral ischemia–reperfusion include strategies encompassing restoration of ionic and metabolic equilibrium, modulation of cell death and neuroinflammatory signaling pathways, structural repair and functional preservation of the BBB and emerging directions for translational research. (Abbreviations: TRPM7: transient receptor potential melastatin 7; MMP: matrix metalloproteinases; TJ: tight junction; VEGF: vascular endothelial growth factor; EPC: endothelial progenitor cell; BBB: blood–brain barrier; ECM: extracellular matrix. Arrow indicators: → indicates pathological cascade and sequential progression; ⊣ indicates inhibition and blockade.)

The temporal sequence outlined above has critical implications for therapy. Interventions that target ROS bursts may be ineffective if administered beyond the 6 h window; conversely, MMP inhibition, beneficial at 12–48 h, may impair repair if continued into the recovery phase. Future therapeutic strategies should consider phase-specific, time-limited interventions rather than sustained blockade of any single pathway. An effective therapeutic framework should integrate three major intervention pillars (Figure 2):

*Restoration of ion and metabolic homeostasis*—preventing excitotoxicity, Ca^2+^ overload and mitochondrial dysfunction.

*Modulation of cell death and inflammatory pathways*—inhibiting apoptosis, pyroptosis, ferroptosis, oxidative stress and immune-mediated injury.

*Reconstruction and preservation of BBB structure*—maintaining TJ integrity, suppressing MMP activity and promoting endothelial repair and angiogenesis.

Recent advances include small-molecule inhibitors, biologics, gene modulation strategies and nanocarrier-based delivery systems targeting these processes [69,70,71]. While these approaches aim to reduce acute BBB disruption and support long-term vascular remodeling, their clinical utility remains limited. These gaps stem from inadequate consideration of evidence limitations and failure drivers, as discussed below.

### 4.1. Restoration of Ion and Metabolic Homeostasis

As mentioned in Section 3.1, the first pathological change is disruption of ion balance, followed by metabolic energy disorders, which may lead to subsequent dysfunction of the BBB. Restoring ion and metabolic homeostasis aims to interrupt the primary pathological cascade before irreversible injury occurs, yet clinical translation of this class of strategies has been hindered by target selectivity, brain penetration, and model–reality mismatches.

#### 4.1.1. Regulation of Ion Channels/Exchangers

Targeting TRPM7 is theoretically attractive for BBB protection, but no TRPM7-targeted drug has entered stroke clinical trials due to insufficient selectivity for TRPM7 homologs and poor BBB penetration. TRPM7 is a unique dual-function protein that acts as both an ion channel permeable to Ca^2+^ and Mg^2+^ and a kinase. During ischemia, TRPM7 is significantly activated by depolarization of the cell membrane and ROS [20]. Pharmacological inhibition (e.g., waixenicin A or carvacrol) or RNA interference of TRPM7 reduces Ca^2+^ influx, attenuates mitochondrial depolarization and preserves BBB integrity in experimental stroke models [72,73]. Future research must prioritize development of such agents with explicit evaluation of off-target effects to de-risk clinical translation.

NHE inhibitors face a different barrier: unreliable extrapolation from cardiovascular models to stroke. HE inhibitors (e.g., S0859, HOE642) effectively reduce brain edema by absorbing excess Na^+^ and preventing TJ protein loss, and they have been evaluated in clinical trials for cardiovascular diseases [74,75]. Their efficacy in acute ischemic stroke remains untested in humans. Future studies must prioritize stroke-specific preclinical models (e.g., aged or comorbid animals) and early-phase trials designed to evaluate BBB penetration and cerebral efficacy, rather than relying on systemic disease data.

#### 4.1.2. Mitochondrial Protection

Mitochondrial protection has failed in stroke translation not because the target is invalid but because preclinical models over relied on young, healthy animals and supraphysiological drug doses. Cyclosporine A preserves mitochondrial integrity by inhibiting the opening of the mPTP, thereby preventing the release of pro-apoptotic factors [76]. MitoQ functions as a mitochondria-targeted antioxidant, precisely neutralizing excess ROS generated during respiration [77]. The SS-31 peptide enhances mitochondrial stability by reinforcing the inner mitochondrial membrane. Due to the limited therapeutic effect of MitoQ, the short half-life and low bioavailability of SS-31, the clinical transformation in the field of stroke has failed [78,79]. Future mitochondrial-targeted strategies must address these limitations by using clinically relevant models, optimizing drug delivery (e.g., using nanocarriers to extend half-life) and validating efficacy at safe, bioavailable brain doses.

#### 4.1.3. Metabolic Regulation

Metabolic regulators have demonstrated target engagement but failed clinical translation due to toxicity, population selection, or dose-timing challenges, each offering a distinct lesson. Dichloroacetate (DCA) shifts metabolism to aerobic oxidation but failed phase 2 trials due to unacceptable toxicity [80,81]. This reminds us that preclinical safety tests must prioritize doses and administration routes relevant to humans. Ketone bodies, such as β-hydroxybutyrate (β-HB), act as neuroprotective metabolic substrates [82]. A study indicated that β-HB treatment did not produce a lasting effect or neuroprotection in intracerebral hemorrhage [83]. This difference suggests that the clinical “translation” of β-HB requires “better selection” of the target population. Certain interventions upregulate hypoxia-inducible factor-1α (HIF-1α), thereby activating downstream metabolic pathways that promote angiogenesis and adaptive responses to hypoxia [84]. Since HIF-1 can regulate the secretion of various growth factors that exhibit biphasic effects during stroke progression, these factors exert protective effects in the acute phase but show damaging effects in the chronic phase [85]. The protective effect of HIF-1α depends on moderate, transient activation, whereas continuous or excessive activation leads to damage [86]. Precisely controlling the dosage and timing of HIF-1α treatment in stroke patients is currently the greatest challenge.

Despite strong preclinical evidence, all agents targeting ion channels, mitochondria, and metabolism have failed or stalled in clinical translation due to two common themes: (1) BBB penetration is consistently underestimated; (2) timing mismatch—preclinical studies administer agents before or immediately after reperfusion, while clinical patients receive delayed treatment. Future success requires brain-targeted delivery systems and validation in clinically relevant time windows.

### 4.2. Immune Regulation and Oxidative Stress Control

#### 4.2.1. Dual-Acting Agents

Multi-target agents (e.g., melatonin, curcumin, edaravone) have achieved the most advanced clinical translation in this category because they simultaneously address antioxidant, anti-apoptotic and anti-inflammatory pathways, which is particularly attractive for complex CIRI pathophysiology. Edaravone was already approved clinically in Japan for acute ischemic stroke, with demonstrated benefits in reducing edema and BBB damage [87,88,89]. Future development must address bioavailability issues (e.g., nanocarrier delivery) and validate efficacy in diverse patient populations.

#### 4.2.2. Anti-Inflammatory Strategies

Anti-inflammatory strategies have repeatedly failed in stroke trials for two core reasons: most agents target a single cytokine despite the redundant, interconnected nature of neuroinflammation, and non-selective inhibition disrupts the biphasic (acute pro-inflammatory, subacute reparative) inflammatory response. Targeting upstream inflammatory mediators, such as TNF-α (e.g., etanercept), IL-1β (e.g., anakinra) and IL-17 (e.g., secukinumab), can mitigate cytokine-induced TJ breakdown [90,91,92]. Downstream interventions, including blocking adhesion molecules (e.g., anti-ICAM-1 antibodies) or chemokine receptors (e.g., CCR2 antagonists), limit leukocyte infiltration into the CNS [93,94]. This failure underscores that future anti-inflammatory strategies must adopt either multi-target approaches or temporally restricted single-target inhibition that spares the reparative phase.

### 4.3. Inhibition of Excessive Proteolysis and Matrix Remodeling

Protecting and restoring BBB structural integrity in CIRI requires interventions that stabilize TJs, suppress ECM degradation and promote endothelial repair [10]. Such strategies are relevant both in the acute phase, to prevent barrier collapse and in the subacute phase, to facilitate vascular repair. The timing of intervention and balance between protective and reparative mechanisms have proven to be critical, underappreciated factors in translational failure.

#### 4.3.1. MMP Inhibition

Early inhibition reduces acute injury and hemorrhagic transformation, whereas inhibition delayed into the vascular repair phase suppresses neurovascular remodeling and exacerbates injury [95]. Inhibition of these enzymes with agents such as minocycline or selective MMP-9 blockers can reduce BBB leakage and the risk of hemorrhagic transformation [96,97]. Yoshihiro Murata et al. found that delaying the administration of the JNK-specific inhibitor SP600125 during the recovery period, such as the angiogenesis stage, could significantly downregulate MMP-9 expression, thereby inhibiting neurovascular remodeling and exacerbating ischemic brain injury [98]. Future MMP inhibitors must incorporate time-dependent release mechanisms; continuous blockade is detrimental, and clinical trials must stratify patients by time-from-stroke-onset rather than treating the acute and subacute phases identically.

#### 4.3.2. Endothelial Cell Regeneration and Basement Membrane Protection

Strategies to preserve TJ proteins and promote endothelial repair have shown preclinical promise, but their failure to advance to late-phase trials reflects weak evidence strength (in vitro or acute-only models) and outcome measures that prioritize systemic endpoints over cerebrovascular integrity.

Agents that preserve TJ proteins (occludin, claudin-5, ZO-1), Ang1 (TIE2 activation) and statins (pleiotropic anti-inflammatory/eNOS-activating effects) have shown preclinical promise for BBB stabilization [99,100,101]. Natural compounds (resveratrol, baicalin) act synergistically to reinforce TJs, but neither has advanced to late-phase clinical trials [102]. Although statins are widely used for secondary stroke prevention, clinical trials have systematically emphasized systemic cardiovascular endpoints, with minimal attention to cerebrovascular integrity or direct BBB functional outcomes.

VEGF-based therapies require controlled delivery; premature exogenous administration exacerbates vascular permeability despite pro-angiogenic effects [103]. Preclinical studies have not adequately tested delayed treatment regimens. EPC transplantation suffers from limited clinical evidence: small patient cohorts, lack of controls, and unclear mechanisms [104]. Randomized controlled trials are needed. Future EPC research must prioritize randomized controlled trials (RCTs) to validate efficacy and identify patient subgroups most likely to benefit. Basement membrane targeting strategies (e.g., ATN-161 and laminin supplementation) have demonstrated preclinical efficacy [105,106].

For endothelial repair strategies such as VEGF, EPC transplantation, and basement membrane targeting (e.g., ATN-161, laminin), the narrow therapeutic windows observed in preclinical studies (immediate post-reperfusion) are clinically impractical; future research must focus on extending these windows, alongside addressing VEGF-induced permeability, EPC clinical evidence gaps, and ATN-161 timing mismatches.

### 4.4. Inhibition of the Programmed Cell Death Pathway

Inhibitors of apoptosis, pyroptosis, and ferroptosis have consistently shown preclinical efficacy in reducing BBB disruption, but their clinical translation has been uniformly limited by poor brain delivery, invasive administration routes, or model–reality mismatches—not by lack of target engagement.

Caspase inhibitors (e.g., z-VAD-fmk), selective caspase-3 inhibitors and Bcl-2 family modulators have shown preclinical efficacy in reducing BBB disruption [107,108,109]. z-VAD-fmk required an invasive intraventricular injection, which is problematic for translation into clinical practice [110]. More precise gene therapy approaches, such as siRNA-mediated knockdown of the pro-apoptotic protein Bax, have also demonstrated BBB protection in animal models [111]. Although anti-apoptotic agents are highly effective in animals, their clinical translation has been limited by poor brain delivery.

NLRP3 inflammasome inhibitors (MCC950), caspase-1 inhibitors (VX-765) and GSDMD blockers (disulfiram) reduce endothelial pyroptosis [112,113,114]. These agents are especially effective in reperfusion injury, where inflammasome activation is rapid and robust. VX-765 has demonstrated some safety in the treatment of epilepsy; its safety and tolerability in stroke patients still need to be verified through larger-scale clinical trials [115]. Disulfiram, an approved anti-alcoholism drug, is being repurposed in preclinical stroke studies for its GSDMD inhibition [116]. Studies have confirmed that it can alleviate CIRI and exert neuroprotective effects by inhibiting microglial pyroptosis and reducing disulfidptosis-like cytoskeletal and mitochondrial damage [117]. Its repurposing potential is promising, but preclinical studies have not addressed whether its systemic effects interfere with cerebral repair.

Ferroptosis inhibitors (deferoxamine, ferrostatin-1) mitigate endothelial damage [118,119]. The clinical trial of deferiprone in Parkinson’s disease failed despite successful preclinical research, and this was attributed to the fundamental differences between the preclinical models and human diseases. This warning is equally important for ischemic stroke [120]. GPX4 activators (e.g., Huoxue Rongluo formula) enhance the activity of glutathione peroxidase 4, which detoxifies lipid hydroperoxides and further suppresses oxidative membrane injury [121]. Despite the achievements mentioned above, the path to clinical translation remains challenging.

The path forward requires brain-penetrant formulations and clinically feasible administration routes—not more preclinical efficacy studies. Combining ferroptosis inhibitors with antioxidants or MMP inhibitors may yield synergistic effects, but this remains to be tested in clinically realistic models.

### 4.5. Non-Pharmacological Interventions

Non-pharmacological interventions (therapeutic hypothermia, remote ischemic preconditioning, mesenchymal stem cells) all show preclinical BBB protection, but their clinical translation progress varies considerably by intervention.

Therapeutic hypothermia (TH) has the most extensive preclinical evidence across MCAO and hypoxic-ischemic models, supporting its efficacy in reducing apoptosis, oxidative stress, neuroinflammation and preserving BBB integrity [122,123,124]. TH failed phase III trials due to difficulty achieving target temperatures and slow enrollment [125].

Mesenchymal stem cell (MSC) therapy shows potential for functional recovery, but phase 2 trials are small and lack long-term follow-up [126,127,128].

Remote ischemic preconditioning (RIPC) significantly downregulates MMP-9 expression and attenuates BBB hyperpermeability, potentially via JAK2/STAT3 inhibition, but lacks standardized protocols and limited clinical evidence [129,130].

It can be concluded that rigorous clinical trial architecture, standardized operational paradigms and large-scale multicenter patient cohorts are indispensable for efficacy verification of non-pharmacological interventions. Mere sporadic positive findings derived from small-sample investigations are insufficient to support robust clinical evidence.

### 4.6. Summary of Intervention Measures for CIRI-Related BBB Protection in Clinical Trials

Table 2 systematically summarizes the drugs and interventions that have been tested in clinical trials for the prevention or alleviation of reperfusion injury after ischemic stroke. It clearly presents the clinical research progress, mechanisms of action and existing research limitations of various strategies, providing a reference for subsequent research and clinical applications.

## 5. Conclusions and Future Directions

The BBB breakdown after CIRI is a complex pathophysiological process involving many related factors. This is a result of synergy among several processes, including ROS formation, sterile inflammation in the CNS, enzymatic activation of matrix metalloproteinases and various forms of programmed cell death (apoptotic, pyroptotic and ferroptosis-induced cell death). This constellation of pathologic processes forms an amplifying feedback loop that increases vascular permeability, promotes fluid leakage into brain tissue and increases bleeding complications. These multiple interactions make therapy challenging and reduce the efficacy of targeted single-molecule pathway therapies.

While prior reviews have focused on individual mechanisms or single therapeutic classes, this review advances three novel integrative concepts:

Temporal hierarchy of injury—Ion dysregulation (minutes) → ROS generation (minutes to hours) → inflammation (hours to days) → MMP activation (hours to days) → cell death (hours to days). Therapeutic timing must match this hierarchy.

Pathway redundancy—Oxidative stress and inflammation form a self-sustaining loop such that targeting either alone is insufficient; dual or sequential targeting is required.

BBB as therapeutic target, not obstacle—Rather than viewing the BBB as a barrier to drug delivery, preserving BBB integrity is itself a therapeutic endpoint that enables subsequent neuroprotection.

To address these challenges, future research should prioritize the following strategic directions:

Temporal precision—Such approaches as MMP inhibition, controlled VEGF delivery and ferroptosis blockade are needed to balance acute neuroprotection with long-term vascular repair and remodeling. Delayed MMP inhibition may mitigate acute injury while preserving angiogenesis in the subacute phase.

Combination and multi-target strategies—Simultaneously targeting oxidative stress, inflammatory signaling, protease activity and regulated cell death pathways to achieve synergistic BBB protection. This approach aligns with CIRI’s systems-biology nature and addresses the limitations of monotherapies that have historically failed in clinical translation.

Biomarker-guided therapy—Integrating BBB permeability imaging, circulating TJ protein fragments and inflammatory markers to guide treatment initiation, stratify patients and monitor therapeutic response. This precision medicine approach will enable more effective and adaptive therapeutic strategies.

Advanced delivery systems—Employing nanocarriers, targeted biologics and BBB-penetrant prodrugs improve drug localization, enhance brain exposure and minimize systemic side effects. These technologies are critical for overcoming the poor pharmacokinetic properties of many promising BBB-protective agents.

Clinical trial integration—Embedding BBB-protective agents into reperfusion-era stroke management, including thrombolysis and mechanical thrombectomy, can reduce the risk of hemorrhagic transformation and improve functional outcomes. This requires close collaboration between basic scientists, clinicians and regulatory bodies.

Protecting the BBB in CIRI will require a paradigm shift from the traditional focus on single-target neuroprotection to an integrated model of vascular–neuronal protection. This shift acknowledges that BBB preservation is not only a prerequisite for neuronal survival but also a critical determinant of long-term neurological recovery and functional independence.

## Figures and Tables

**Table 1:** Key molecular markers of the BBB and their regulatory mechanisms in CIRI.

Molecular Type	Representative Molecules	Expression Changes	Functions and Mechanisms	Ref.
Excitotoxic molecules	Glu, NMDAR	Accumulation of Glu in the synaptic cleft → Overactivation of NMDA receptors	Neuronal overexcitation → Increased Ca^2+^ influx → Endothelial cell injury.	[19]
Calcium ion-regulated protein	Calpain, TRPM7	Intracellular Ca^2+^ ↑, Calpain activation	Calcium overload leads to mitochondrial dysfunction and cytoskeletal degradation.	[20,21]
Oxidative stress markers	MDA, GSH-Px, SOD	MDA ↑, GSH-Px, SOD ↓	Accumulation of ROS leads to lipid peroxidation.	[22]
Inflammatory factors	IL-1β, NLRP3, TNF-α, cleaved caspase-1	Activation of the NLRP3 inflammasome leads to increased IL-1β release	Activation of pyroptosis-related inflammatory pathways.	[23]
Basal membrane components	Collagen IV, Laminin	Degradation or fracture	MMP9-mediated degradation promotes BBB disruption.	[24]
TJ proteins	ZO-1, Occludin, Claudin-5	Reduced expression, disordered positioning	Maintains TJs between endothelial cells; their degradation increases BBB permeability.	[25]
Cell apoptosis markers	Cleaved Caspase-3, BAX, Bcl-2	Caspase-3 activation, increased BAX/Bcl-2 ratio	Mitochondrial pathway apoptosis (BAX translocation to mitochondria → Cytochrome C release → Caspase cascade reaction).	[26]
Pyroptosis-related molecules	GSDMD, Caspase-1	The formation of GSDMD cleavage products leads to cell membrane perforation	The executioner protein of pyroptosis (GSDMD) mediates cell lysis and exacerbates BBB permeability.	[27]
Ferroptosis markers	GPX4, ACSL4, PTGS2	GPX4 ↓, ACSL4 ↑, lipid ROS ↑	Iron-dependent lipid peroxidation leads to endothelial cell death.	[28]

Abbreviations: NLRP3: NOD-like receptor thermal protein domain associated protein 3; IL-1β: interleukin-1β; GSDMD: gasdermin D; TNF-α: tumor necrosis factor-α; GPX4: glutathione peroxidase 4; ACSL4: Acyl-CoA synthetase long-chain family member 4; PTGS2: prostaglandin-endoperoxide synthase 2; MDA: malondialdehyde; GSH-Px: glutathione peroxidase; SOD: superoxide dismutase; TRPM7: transient receptor potential melastatin 7; NMDAR: N-methyl-D-aspartate receptor; Glu: glutamate; BAX: BCL2-associated X protein; Bcl-2: B-cell lymphoma 2; BBB: blood–brain barrier; MMP9: matrix metalloproteinase 9. Arrow indicators: ↑ indicates significant increase/upregulation; ↓ indicates significant decrease/downregulation; → indicates pathological cascade and sequential progression.

**Table 2:** Drugs/interventions that have been verified in clinical trials for the prevention or alleviation of reperfusion injury after ischemic stroke.

Category	Drug	Conclusion	Critical Analysis & Lessons for Future Research	Clinic Trail	Ref.
Restoration of ion and metabolic homeostasis	Cyclosporine A	It failed to reduce infarction size effectively and studies have shown no benefit.	Failure attributed to poor BBB penetration and a mismatch between preclinical (young animals) and clinical (aged/comorbid patients) models. Future mitochondrial-targeted agents must prioritize brain delivery and validate in clinically relevant models.	Phase II clinical trial	[131,132]
	Nerinetide	It could slightly reduce the mortality rate of AIS, but functional outcomes were not significantly improved.	A modest benefit reflects a narrow therapeutic window and incomplete targeting of excitotoxicity. Future excitotoxicity inhibitors should combine with other pathways (e.g., anti-inflammation) to enhance efficacy.	Phase III clinical trial	[133]
	Magnesium sulfate	Ultra-early administration was safe but did not have a significant neuroprotective effect.	Failure due to delayed administration in most patients (beyond the ultra-early window) and inadequate consideration of comorbidities. Highlights the need for extended therapeutic windows or patient stratification by treatment timing.	Phase III clinical trial	[134]
	Dextromethorphan	Safe, but with no significant neuroprotective benefits; it was not advanced to Phase III and has limited clinical application.	Lack of efficacy reflects weak preclinical evidence (overreliance on acute models) and poor BBB penetration. Future agents must validate efficacy in chronic models and optimize delivery.	Phase II clinical trial	[135]
	Aptiganel/ Cerestat	The high-dose group had a higher mortality rate, obvious central adverse reactions and no neuroprotective benefits.	Failure due to poor dose optimization and off-target effects. Emphasizes the need for preclinical dose–response studies and rigorous safety testing before Phase III.	Phase III clinical trial	[136]
	Selfotel	The therapeutic effect is not definite, and it may also have neurotoxic effects in cerebral ischemia.	Neurotoxicity is likely due to non-selective glutamate receptor targeting. Future excitotoxicity inhibitors must be subtype-specific to avoid off-target harm.	Phase III clinical trial	[137]
Modulation of cell death and inflammatory pathways	Edaravone dexborneol	Its therapeutic effect was superior to that of edaravone, but its safety issues were a concern.	Relative success due to multi-target (antioxidant, anti-inflammatory) mechanism. Safety concerns highlight the need for long-term follow-up in diverse patient populations.	Phase III/IV clinical trials	[138,139]
	Cerovive	Failure was declared, as no positive results could be replicated.	Failure due to inconsistent preclinical reproducibility and poor trial design. Highlights the need for rigorous preclinical validation before clinical testing.	Phase III clinical trial	[140]
	Alpha-lipoic acid	It was in the exploration stage, and no clear evidence of clinical benefits has emerged yet.	Limited evidence due to small sample size and lack of BBB penetration data. Future trials should measure cerebral drug concentrations and use larger cohorts.	Phase II exploratory clinical trial	[141]
	Minocycline	Effectiveness in improving patients’ functional prognosis and ensuring good safety.	Success attributed to multi-target effects (MMP inhibition, anti-inflammation) and good BBB penetration. Serves as a model for future multi-target agents.	Phase III clinical trial	[142]
	glibenclamide	The primary endpoint was not reached, and there was no significant improvement in functional outcomes.	Failure due to oversimplification of pathophysiology and delayed administration. Future strategies should combine with other pathways.	Phase III clinical trial	[143]
	Fingolimod	It has good safety and can improve early neurological deficits.	Promising early results, but limited by a small sample size. Future trials should focus on patient stratification (e.g., by stroke severity) to identify responders.	Phase II exploratory clinical trial	[144,145]
	Natalizumab	The primary endpoint was not reached, and there was no significant improvement in functional outcomes.	Failure due to non-selective inhibition of leukocyte infiltration, which disrupts reparative processes. Highlights the need for context-specific anti-inflammatory targeting (acute vs. subacute).	Phase II clinical trial	[146]
	Anakinra	Preliminary exploration of anti-inflammatory effects has been conducted, but no clear evidence of reperfusion injury protection has been formed yet.	Limited benefit due to targeting a single cytokine (IL-1β) in a redundant inflammatory network. Future anti-inflammatory strategies should target multiple mediators or upstream pathways.	Phase II clinical trial	[147]
	DL-3-n-butylphthalide	It significantly improved neurological deficits and has good safety, making it a first-line drug for acute ischemic stroke in China.	Success due to multi-target mechanisms (antioxidation, anti-inflammation, BBB protection) and optimization for Chinese patient populations. Demonstrates the value of population-specific drug development.	Phase III/IV clinical trials	[148,149]
	deferoxamine	Dose exploration and safety trials were lacking in confirmatory efficacy studies.	Limited evidence due to inadequate dose optimization. Future ferroptosis inhibitors must prioritize dose–response studies to balance efficacy and safety.	Phase II clinical trial	[150]
	Otaplimastat	Intravenous administration of oplimaristat as an adjuvant treatment for patients receiving rt-PA was feasible and generally safe.	Safety established, but efficacy unclear due to small sample size. Future trials should evaluate efficacy in combination with rt-PA, focusing on BBB leakage endpoints.	Phase II clinical trial	[151]
	Human Urinary kallidinogenase	It effectively improved perfusion in ischemic areas and neurological function and was used in China for reperfusion injury in AIS.	Success is attributed to the targeted improvement of cerebral microcirculation. Highlights the value of strategies that address BBB dysfunction indirectly via microvascular repair.	Phase IV clinical trial	[152]
Regulation of microcirculation, anticoagulation and thrombolysis	Cilostazol	For secondary prevention of non-cardiogenic ischemic stroke.	Success in secondary prevention, but no role in acute BBB protection. Emphasizes the need to distinguish between acute and chronic therapeutic goals.	Phase IV clinical trial	[153]
	rt-PA	Significantly improved prognosis.	Gold standard for thrombolysis, but may exacerbate BBB leakage in some patients. Future research should combine rt-PA with BBB-stabilizing agents to mitigate risk.	Phase III clinical trial	[154,155]
	Tenecteplas	The recanalization rate was higher than that of alteplase, and the safety was comparable.	Superior recanalization, but similar BBB leakage risk to rt-PA. Combination with TJ stabilizers may further improve outcomes.	Phase III clinical trial	[155,156]
	Tirofiban	Early use was associated with good functional outcomes, but the risk of bleeding increased.	Balancing efficacy (functional improvement) and safety (bleeding risk) is critical. Future trials should stratify patients by bleeding risk to optimize use.	Phase III clinical trial	[157]
	Eptifibatide and Argatroban	Negative outcome; failed to demonstrate functional benefit	Failure due to inadequate targeting of microcirculatory dysfunction and increased bleeding risk. Highlights the need for more precise anticoagulant/antiplatelet strategies in acute stroke.	Phase III clinical trial	[158]
Physical intervention and stem cell regeneration	Remote ischemic preconditioning	Exploratory application, no standardized protocol has been formed, and clinical evidence is limited.	Limited progress due to lack of standardized protocols (e.g., ischemia duration, number of cycles). Future research must establish consensus on treatment parameters before large-scale trials.	Clinical exploratory research	[159,160]
	Therapeutic hypothermia	Only a portion of the patients reached the target temperature, and there was no difference in the efficacy analysis. Additionally, the trial was terminated due to slow enrollment and financial issues.	Failure due to logistical challenges (target temperature maintenance) and poor trial design. Future hypothermia studies should optimize delivery (e.g., endovascular cooling) and improve enrollment strategies.	Phase III clinical trial	[125]
	Hyperbaric oxygen therapy	It was used as an adjuvant treatment for reperfusion injury, and its therapeutic effect shows individual differences, warranting further experimental research.	Variable efficacy due to patient heterogeneity (e.g., stroke severity, treatment timing). Future trials should stratify patients to identify responders.	Phase II clinical trial	[161]
	Mesenchymal stromal cell	It may improve functional recovery, but its therapeutic effect has not been fully confirmed.	Limited evidence due to small cohorts and unclear mechanisms. Future studies should focus on cell source, dose and administration route, with RCTs to validate efficacy.	Phase II clinical trial	[128]
	Endothelial progenitor cell	Intracarotid infusion of autologous CD34^+^ cells was safe and may improve the long-term prognosis of patients with acute ischemic stroke.	Promising safety data, but efficacy requires larger RCTs with long-term follow-up. Highlights the potential of cell-based therapies for chronic BBB repair.	Phase II clinical trial	[162]

## Data Availability

No new data were created or analyzed in this study.

## Abbreviations

The following abbreviations are used in this manuscript:

CIRI	Cerebral Ischemia–Reperfusion Injury
MCAO	Middle Cerebral Artery Occlusion
BMEC	Brain Microvascular Endothelial Cells
MPTP	Mitochondrial Permeability Transition Pore
BBB	Blood–Brain Barrier
ACSL4	Acyl-CoA Synthetase Long-chain Family Member-4
LOX15	Arachidonate 15-Lipoxygenase
NLRP3	NOD-like Receptor Thermal Protein Domain Associated Protein-3
TJ	Tight Junction
CNS	Central Nervous System
ANLS	Astrocyte Neuron Lactate Shuttle
MMP-9	Matrix Metalloproteinases-9
GSDMD	Gasdermin D
DAMPs	Damage-Associated Molecular Patterns
PRRs	Pattern Recognition Receptors
